# Synthesis and Characterization of Methyl Cellulose/Keratin Hydrolysate Composite Membranes

**DOI:** 10.3390/polym9030091

**Published:** 2017-03-04

**Authors:** Bernd M. Liebeck, Natalia Hidalgo, Georg Roth, Crisan Popescu, Alexander Böker

**Affiliations:** 1 Institut für Kristallographie, RWTH Aachen University, Jägerstr. 17-19, D-52066 Aachen, Germany; roth@ifk.rwth-aachen.de; 2 DWI—Leibniz-Institut für Interaktive Materialien e.V., Forckenbeckstr. 50, D-52074 Aachen, Germany; natalia.hidalgo@rwth-aachen.de; 3 KAO European Research Laboratory, KAO Germany GmbH, Pfungstadterstr. 98-100, D-64297 Darmstadt, Germany; crisan.popescu@kao.com; 4 Fraunhofer-Institut für Angewandte Polymerforschung (IAP), Geiselbergstr. 69, D-14476 Potsdam-Golm, Germany; alexander.boeker@iap.fraunhofer.de; 5 Lehrstuhl für Polymermaterialien und Polymertechnologien, Universität Potsdam, Geiselbergstr. 69, D-14476 Potsdam-Golm, Germany

**Keywords:** bio-based, composite materials, methyl cellulose, keratin, superheated water

## Abstract

It is known that aqueous keratin hydrolysate solutions can be produced from feathers using superheated water as solvent. This method is optimized in this study by varying the time and temperature of the heat treatment in order to obtain a high solute content in the solution. With the dissolved polypeptides, films are produced using methyl cellulose as supporting material. Thereby, novel composite membranes are produced from bio-waste. It is expected that these materials exhibit both protein and polysaccharide properties. The influence of the embedded keratin hydrolysates on the methyl cellulose structure is investigated using Fourier transform infrared spectroscopy (FTIR) and wide angle X-ray diffraction (WAXD). Adsorption peaks of both components are present in the spectra of the membranes, while the X-ray analysis shows that the polypeptides are incorporated into the semi-crystalline methyl cellulose structure. This behavior significantly influences the mechanical properties of the composite films as is shown by tensile tests. Since further processing steps, e.g., crosslinking, may involve a heat treatment, thermogravimetric analysis (TGA) is applied to obtain information on the thermal stability of the composite materials.

## 1. Introduction

The utilization of bio-based materials is an efficient approach to reduce the negative impacts of petroleum-based products such as CO_2_ emission and global pollution. Therefore, biomaterials from renewable resources such as proteins or polysaccharides are increasingly gaining interest since they are considered as renewable, environmentally friendly, non-toxic, biocompatible, biodegradable and inexpensive materials [1]. One of the most abundant proteins in nature is keratin. As feathers contain around 90 w% keratin, they are an attractive material for obtaining this insoluble and highly durable protein [2]. Huge amounts of feathers are produced as a byproduct of the poultry industry; most of them are burned in waste incinerating plants, disposed of in landfills or recycled into low quality animal feeds. Thereby a potentially valuable resource is wasted, and certain environmental problems are caused [2,3,4,5].

Whole feathers were used recently, e.g., as reinforcement in light weight composites [3,4] or extruded fibers [6]. In addition, solubilized keratin is becoming more frequently used as a source of biomaterials in compostable packaging and regenerated fibers [6], since it is biocompatible, biodegradable and possesses better gas barrier properties compared to polysaccharides and lipids [7,8]. For this purpose, the molecular bonds between the proteins must be broken down while keeping the strands intact or at least breaking them to a controlled level. Keratin is rich in cysteine residues and the molecules form a three-dimensional polymer interlinked by disulfide bonds. This, supported by the high amount of hydrophobic amino acids, leads to the insolubility of keratin in both, polar and nonpolar solvents. Thus, most dissolution routes require a variety of—partly hazardous—chemicals [6,7,9], are time consuming and require subsequent processes in order to remove the excess chemicals. It has been shown that the treatment of feathers with superheated water (H_2_O heated under pressure above its atmospheric boiling point) converts this keratin source in solution by breaking the keratin chains into polypeptides of 10 to 20 amino-acids [10,11]. These strands are comparatively short, and result in materials with inferior properties. However, by blending these polypeptides with a matrix material, the mechanical properties can be significantly enhanced, resulting in a novel composite material which combines the properties of the protein and matrix.

A promising candidate for this task is the most common organic polymer in the world, cellulose. This polysaccharide is considered as an almost inexhaustible source for environmentally friendly materials [12]. It is abundant, renewable, biodegradable as well as biocompatible [13,14] and used in a wide range of applications, from paper, regenerated films and fibers [12] to various medical applications [13]. Cellulose can easily be chemically modified, e.g., into cellulose esters or ethers [15] which possess good film-forming properties such as transparency, flexibility and resistance to oil and fats [16]. Methyl cellulose, one of the most widely produced cellulose derivatives [15], possesses, in addition to these properties, a good solubility in aqueous solutions.

Thus, by mixing aqueous solutions of keratin polypeptides—obtained through solubilizing feather keratin in superheated water—with solutions of methyl cellulose, it is feasible to cast composite membranes. These films, produced by the reuse of bio-waste, are distinguished by the combined properties of proteins and polysaccharides. The existence of polypeptides among cellulose chains, for example, might provide sites enhancing a specific ‘breathing motion’ (fluctuation in the three-dimensional structure) like proteins do. Hence, the structure–properties relationship and the implications at the mesoscopic and macroscopic level between keratin-based polypeptides and cellulose derivatives are the subject of the present work. The interactions between the components are investigated by Fourier transform infrared spectroscopy (FTIR), wide angle X-ray diffraction (WAXD), thermal gravimetric analysis (TGA) and tensile testing. The acquired data may help to clarify the role of the interaction between polypeptides and polysaccharides for the stability of the material and to describe the mechanical and chemical behavior of keratin-based hybrid materials. This offers, on the one hand, a theoretical background for further studies on the stability of protein-based materials and, on the other hand, it gives a support for designing new hybrid biopolymers based on keratin.

## 2. Materials and Methods

### 2.1. Materials

Commercially available cleaned and degreased mixtures of feathers and down in a mixing ratio of 70/30 of the domestic goose (*Anser anser domesticus*)—as used for pillow fillings—were employed as starting material. For preparation of the keratin hydrolysate solutions, feathers were solubilized in deionized water as described below. Methyl cellulose (viscosity 400 cP, 2% in H_2_O, 27.5%–31.5% methoxyl) and glycerol (purity 99%) both from Sigma-Aldrich (Schnelldorf, Germany) were used as received without further purification.

### 2.2. Solubilization of Goose Feathers

Goose feathers (2–5 mg) were weighed and placed in stainless steel autoclaves with Teflon inlays. Afterwards, the required amount of deionized water was added. The vessels were sealed and placed in a preheated oven. After the extraction experiment, the autoclaves were cooled down to room temperature in a fume hood. The obtained keratin hydrolysate solutions were filtered with a filter paper in order to remove non-dissolved particles and stored in glass flasks at room temperature.

In order to evaluate the dissolution of the feathers, several process parameters were varied. Initial feather to water concentrations of 20, 50, 100 and 250 mg·mL${}^{-1}$ were investigated. Oven temperatures of 180, 200 and 220 °C were used as well as heating times of 30, 60 and 120 min for each combination. The dried, non-dissolved residues were weighted and thereby the yield *η* of the dissolved feathers calculated by

(1) $$\eta =\frac{m_{f}-m_{r}}{m_{f}}\cdot 100\%$$

where $m_{f}$ is the initial weight of the utilized feathers and $m_{r}$ the weight of the dried, non-dissolved deposits after the extractions.

### 2.3. Film Preparation

**Figure 1:**
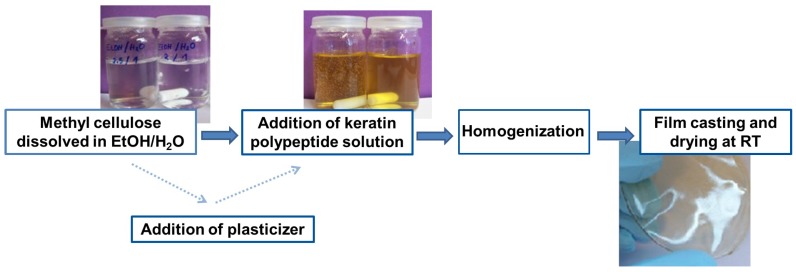
Schematic presentation of the film preparation.

The utilized procedure for the film preparation is visualized in Figure 1. A concentration of 20 mg·mL${}^{-1}$ methyl cellulose powder was dissolved in an ethanol–water solution (2.5:1 *v*/*v*). Under permanent stirring, the mixture was homogenized for 1.5 h at 65 °C until a colorless, viscous solution was obtained.

If glycerol as plasticizer was added, the solution was homogenized for another 30 min. Thereafter, the prescribed amount of polypeptide solution was admixed. The added keratin hydrolysate solution, used for the film forming process, was obtained as described by dissolving 250 mg·mL${}^{-1}$ goose feathers in deionized water for 120 min at 220 °C. The mixture of methyl cellulose and polypeptide solution was stirred for another 30 min to ensure a homogeneous distribution. Subsequently the cooled mixture was poured into Petri dishes, covered with paper towels and air dried for at least 48 h in a fume hood at room temperature. The obtained films were peeled off from the glass and stored in ambient conditions.

### 2.4. Variation of the Keratin Hydrolysate Amount in the Films

In order to investigate its influence on the properties of the hybrid films, the amount of keratin hydrolysate solution (250 mg·mL${}^{-1}$, 120 min, 220 °C) added to the methyl cellulose solution (20 mg·mL${}^{-1}$) was varied as described in Table 1. The methyl cellulose to keratin hydrolysate (MC/Ker) ratio in the dried films was calculated by

(2) $$MC/Ker=\frac{dwt_{MC}}{dwt_{Ker}}=\frac{V_{MC}\cdot 20\;mg/mL}{V_{Ker}\cdot 250\;mg/mL\cdot 67\%}$$

where $dwt_{MC}$ and $dwt_{Ker}$ is the calculated dry weight of methyl cellulose or keratin hydrolysates respectively, $V_{MC}$ is the volume of the used methyl cellulose solution (containing 20 mg·mL${}^{-1}$ methyl cellulose) and $V_{Ker}$ the added amount of polypeptide solution with an initial feather to water ratio of 250 mg·mL${}^{-1}$ (processing parameters: 120 min at 220 °C). The solubility of the feathers (yield *η*) for the chosen process parameters was experimentally determined by Equation (1) and found to be 67% (see Table 2).

Films investigated with FTIR and WAXD were produced without further additives as the interaction between the methyl cellulose and the keratin hydrolysates should be investigated. For tensile tests and thermal analysis, 25 μL glycerol as plasticizer was added to the film casting solution in order to improve the mechanical properties of the tested materials.

### 2.5. Fourier Transform Infrared Spectroscopy (FTIR)

Fourier transform infrared spectroscopy (FTIR) measurements were carried out directly on the films. A Nicolet iS10 FTIR spectrometer (Thermo Fisher Scientific, Waltham, MA, USA) at the Institut für Textiltechnik (ITA), RWTH Aachen University equipped with a diamond attenuated total reflectance (ATR) device was utilized. A wave number range from 550 cm${}^{-1}$ to 4000 cm${}^{-1}$ was recorded with a resolution of 4 cm${}^{-1}$; 36 scans per sample were performed and averaged. The obtained spectra were baseline-corrected using the OMNIC Specta software and normalized on the band appearing at 1053 cm${}^{-1}$ for better comparison.

### 2.6. Wide Angle X-Ray Diffraction (WAXD)

Wide angle X-ray diffraction (WAXD) experiments on dried keratin hydrolysates (250 mg·mL${}^{-1}$, 220 °C, 120 min) and composite films containing methyl cellulose and keratin polypeptides were carried out in reflection geometry using a Philips X’Pert Pro powder X-ray diffractometer equipped with a Ni filter and CuK$\alpha_{1,2}$ radiation (*λ* = 1.541 Å). Polypeptide solutions were dried in a Petri dish at ambient conditions, scratched off the glass and pestled carefully to ensure homogeneity. From the films, pieces were cut out for investigation. The samples were placed on a low background silicon single crystal sample holder and measured in a 2*θ* range from 5° to 60° with a step size of 0.008° and a counting time of 40 s per step.

Intensities of the obtained WAXD patterns were normalized to the intensity of the first peak for better comparison. Peak positions were obtained through the zero crossing of the first derivative of the normalized and smoothed WAXD patterns. All mathematical operations were performed using OriginPro 9.1 (Origin Lab Corporation) software.

### 2.7. Mechanical Characterization

Mechanical properties of the films were measured using a tensile tester (Minimat Stemi 2000-C). Films including 25 μL glycerol as plasticizer were cut into 2.2 cm long and 1.2 cm wide rectangles. The film thickness was determined with a digital micrometer.

The initial distance between the tensile clamps was set to 10 mm while for the crosshead a speed of 4 mm·min${}^{-1}$ was chosen. Yield strength and ultimate elongation at break were examined, and the Young’s modulus was determined from the 2% offset slope of the initial linear part of the stress–strain curve. At least three measurements for each set of samples were performed, and average values and standard deviations calculated.

### 2.8. Thermogravimetric Analysis (TGA)

Thermogravimetric analysis (TGA) was performed on a PerkinElmer STA 6000 with heating rates of 10 K·min${}^{-1}$. Nitrogen was used as purge gas with a flow rate of 20 mL·min${}^{-1}$. Around 5–10 mg sample mass was heated in open alumina crucibles from room temperature to 650 °C. The first derivative of the TG signal (differential thermogravimetric curve (DTG)) was calculated. The analyzed keratin hydrolysate resulted from a treatment at 220 °C for 120 min with an initial feather to water ratio of 250 mg·mL${}^{-1}$. The characterized films were produced as described above and consisted of methyl cellulose, keratin polypeptides, and 25 μL glycerol as plasticizer.

## 3. Results and Discussion

### 3.1. Solubilization of Goose Feathers

It is known that by using superheated water, feathers from chicken and goose can be decomposed into water soluble polypeptides with a molecular weight around 1–1.8 kDa [10,11]. To obtain more information on the dissolution process, heating temperature, dwell time, and initial feather to water ratio were varied. Afterwards, the amount of dissolved material (Table 2) was determined using Equation (1). Since the composition of the polypeptide solutions has significant influence on the membrane properties, it is important to optimize the process parameters in order to generate the highest possible polypeptide content.

The variation of the process parameters shows that dwell time and temperature have a noticeable effect on the solubility of the feathers. Short dwell times around 30 min are not suitable to dissolve the feather material. Yields below 5% are obtained independent of temperature and initial feather to water concentration; the obtained solutions remain colorless. Heating times of 60 min lead to a significant increase of the polypeptides in the solution. The portion of dissolved material increases considerably with the temperature. Thereby distinct signs of the reaction between feathers and superheated water become noticeable. A smell of H_2_S is recognizable when the autoclaves are opened, indicating the destruction of disulfide bonds. The solutions turn slightly brownish, getting darker with temperature. Leftovers from the (original white) feathers form brown, agglutinated lumps. By increasing the dwell time to 120 min, the highest yields can be achieved for all investigated initial concentrations and temperatures. However, once again the influence of the temperature on the solution behavior of the feathers becomes clear, since the largest amount of material (up to 76% of the initial mass) is dissolved at 220 °C and a dwell time of 120 min. Only few parts of the feathers withstand the heat treatment at high temperatures and long holding times. The non-dissolved particles form a black-brownish sedimentary deposition on the bottom of the Teflon inlay, while the produced solutions are of dark brown color. Presumably, higher temperatures and longer heating times may further increase the amount of dissolved material.

Regarding the initial feather to water ratios, it appears that lower starting concentrations result in higher yields. However, by calculating the solute content for the liquids, it can be seen that a higher absolute amount of initial feathers leads to more dissolved material per mL water. For example, at an initial feather content of 250 mg·mL${}^{-1}$ and a solubility of 67%, a polypeptide concentration of 167.5 mg·mL${}^{-1}$ is obtained in the solution, while an initial feather to water ratio of 20 mg·mL${}^{-1}$ with a solubility of 76% results in a polypeptide concentration of 15.2 mg·mL${}^{-1}$. A high polypeptide concentration in the solution is desirable in order to incorporate a large proportion of the protein component into the membranes. For this reason, polypeptide solutions synthesized at 220 °C for 120 min with an initial concentration of 250 mg·mL${}^{-1}$ are used for the film production.

### 3.2. Keratin Hydrolysate Film Forming Properties

The interaction between the protein- and the polysaccharide-based component is investigated by systematically changing the incorporated amount of polypeptides. Various amounts of polypeptide solution are added to the ethanol/water mixture containing 20 mg·mL${}^{-1}$ methyl cellulose. The solutions are miscible in all investigated ratios, resulting in homogeneous mixtures without any traces of precipitates. Films are casted from these solutions, resulting in calculated methyl cellulose to keratin hydrolysate (MC/Ker) ratios of 300/83.75 (*MC/Ker-1*), 300/167.5 (*MC/Ker-2*), 300/251.25 (*MC/Ker-3*), 300/335 (*MC/Ker-4*), and 300/418.75 (*MC/Ker-5*), as described above (Equation (2)).

**Figure 2:**
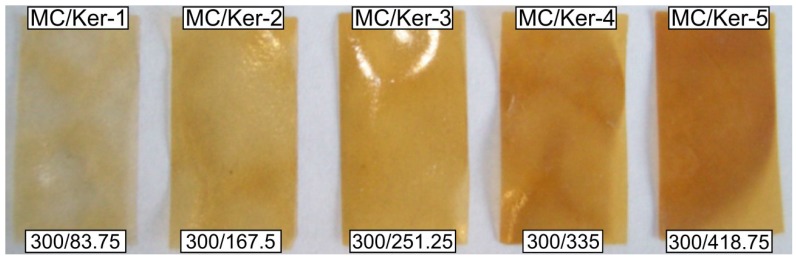
Composite films produced from solutions with different methyl cellulose/keratin hydrolysate (MC/Ker) ratios. Glycerol (25 μL) was added as plasticizer to the solutions for the film casting.

While the pure polypeptides form brittle layers that strongly adhere to the glass substrate, the novel composite membranes, depicted in Figure 2, can be detached easily and undamaged from the carrier material. All films can be cut into pieces without disruption and large areas are free from macroscopic defects such as gas bubbles.

The surface appearance is, independent of the polypeptide concentration, smooth. Film thickness increases with ascending keratin hydrolysate content. While films made from pure methyl cellulose are known for their transparent appearance, the hybrid materials are brown in color, getting darker with increasing amount of polypeptides. Based on these optical observations, no phase separation takes place during the drying process, indicating an interaction between the two components.

### 3.3. FTIR Analysis of the Hybrid Films

**Figure 3:**
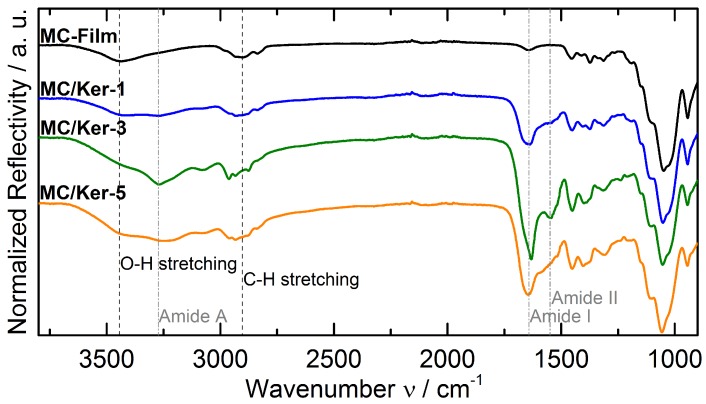
Fourier transform infrared spectroscopy (FTIR) measurements of films with different methyl cellulose/keratin hydrolysate (MC/Ker) ratios.

For further information on how the polypeptides interact with the cellulose derivative, FTIR measurements are carried out. In Figure 3, the spectra of the composite membranes are displayed. A film consisting of pure methyl cellulose is used as reference sample whose absorption peaks can be assigned to peaks known from literature [17,18,19,20]. This includes, among others, the bands for the O–H stretching vibration at approximately 3444 cm${}^{-1}$ and the C–H stretching vibration around 2903 cm${}^{-1}$.

In the FTIR spectra of the composite membranes, typical amide vibrations resulting from the embedded polypeptides appear. This includes, for example, the N–H stretching vibraion of the amide A band at 3273 cm${}^{-1}$ [21,22]. The strong absorption band observed between 1600 and 1700${}^{-1}$ can be attributed to the C=O stretching of the amide I vibration [21,22], while the signal corresponding to the amide II vibration (C–N stretching and N–H bending) between 1480 and 1580 cm${}^{-1}$ [21,22] is noticeably weaker, in some samples only present as a shoulder next to the amide I vibration band. This is in accordance with results obtained from the FTIR studies of the pure keratin hydrolysate, as performed in [11]. This phenomenon is explained by the assumption that most of the hydrogen bonds between the polypeptide chains in the original feathers are broken by the superheated water treatment [11]. However, the presence of the amide bands in the spectra of the composites shows that keratin-based polypeptides are incorporated into the polymer matrix consisting of intact methyl cellulose.

### 3.4. WAXD Analysis of the Hybrid Films

**Figure 4:**
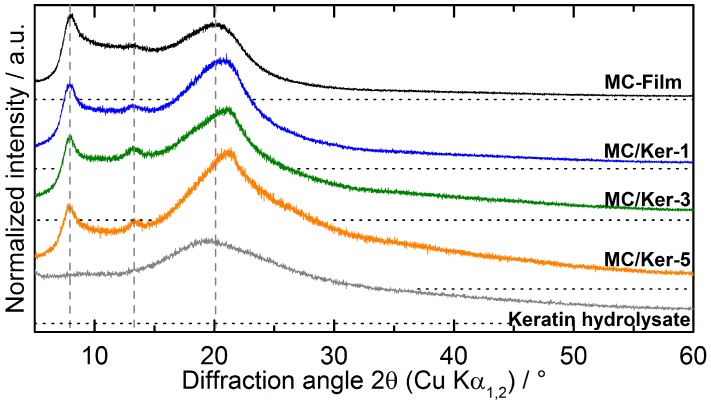
Wide angle X-ray diffraction (WAXD) patterns of composite membranes with various methyl cellulose/keratin hydrolysate (MC/Ker) ratios. The dashed vertical lines mark the peak position for the neat methyl cellulose film, while the dotted horizontal lines give the zero intensity of each diffraction pattern.

Due to the semi-crystalline structure of methyl cellulose, X-ray examinations can reveal how the incorporation of polypeptides affects the biopolymer matrix. WAXD patterns of composite membranes as well as pure keratin hydrolysates are depicted in Figure 4. A reference film casted from neat methyl cellulose exhibits a pattern as expected for this cellulose derivative [20,23]. A broad peak at a diffraction angle 2*θ* (CuK$\alpha_{1,2}$) of 8.03° is known to correspond to the trimethylglucose repeating unit of methyl cellulose [24], while another reflection appears as a broad hump at a diffraction angle of 19.85°. Furthermore, a weak peak appearing at 13.21° 2*θ* indicates a more hydrated structure [25] in the films compared to the as-received powder, where this peak is not present.

In contrast to the highly oriented diffraction pattern of avian keratin in feathers [26] the WAXD pattern of dried keratin hydrolysates consists only of a broad hump with a maximum at a diffraction angle around 19°.

**Figure 5:**
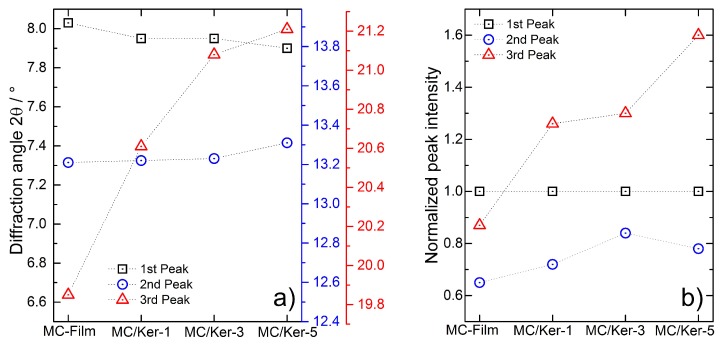
(**a**) Diffraction angle 2*θ* (CuK$\alpha_{1,2}$) of the peaks in the WAXD patterns of the composite membranes. Dotted lines are visual guides; (**b**) Peak intensities (normalized to the intensity of the first peak) in the WAXD patterns of the composite membranes. Dotted lines are visual guides.

The WAXD patterns obtained from the composite membranes are comparable to the pattern of the neat methyl cellulose film. The first peak is present in all investigated compositions with a 2*θ* position between 7.90 and 7.95°, close to that of neat methyl cellulose. The presence of the second peak between 13.21 and 13.31° in all investigated membranes proves that the developed film casting route in general leads to a more hydrated methyl cellulose structure. In contrast to the first two peaks—whose position remains almost unchanged—the third peak in the WAXD patterns of the composite films is distinctly influenced by the addition of the polypeptides. Its position shifts systematically from 19.85° for the neat methyl cellulose film up to a diffraction angle of 21.21° for sample MC/Ker-5 as depicted in Figure 5a. Furthermore, the peak shape becomes more asymmetric with increasing keratin hydrolysate fraction, as shown in the WAXD patterns in Figure 4.

Regarding the normalized intensities (normalized to the intensity of the first peak) as displayed in Figure 5b, it can be seen that the proportion between first and third peak increases significantly from 0.87 to 1.6 with ascending polypeptide fraction. The ratio between the first and second peak remains nearly unchanged.

The systematic changes in peak position and intensity of the methyl cellulose WAXD reflection at 19.85° with a growing amount of added keratin polypeptides prove the incorporation of the keratin hydrolysates into the semi-crystalline methyl cellulose structure. Thus, the two compounds interact at the molecular scale.

### 3.5. Mechanical Characterization of the Hybrid Films

**Figure 6:**
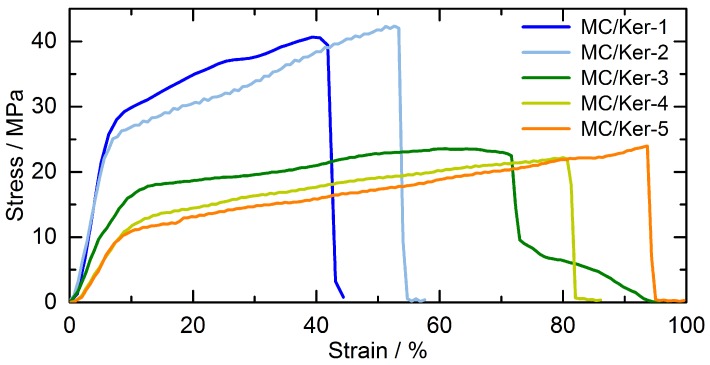
Stress–strain diagrams of composite membranes with various methyl cellulose/keratin hydrolysate (MC/Ker) ratios. Glycerol (25 μL) was added as plasticizer to the solutions for the film casting.

Since the X-ray analysis shows that the semi-crystalline structure of the methyl cellulose matrix is strongly influenced by embedding the keratin hydrolysates, it is of interest to investigate the influence of these molecular interactions on the macroscopic properties of the membranes. Thus, tensile tests were performed and the obtained stress–strain curves for films with different polypeptide contents are displayed in Figure 6. As the incorporation of plasticizers is known to improve the mechanical properties of polymeric materials, 25 μL glycerol was added to the film casting solutions.

From these measurements, Young’s modulus, offset yield strength and ultimate elongation are determined as displayed in Table 3. These results reveal that the mechanical properties change significantly with increasing polypeptide fraction. The offset yield strength decreases remarkably while the ultimate elongation at break increases significantly with the amount of keratin hydrolysates.

The stiffness of the materials decreases, and the films become more flexible and less brittle by adding the keratin-based polypeptides. As shown by X-ray diffraction, the keratin hydrolysates influence the crystalline structure of the methyl cellulose. Thus, they probably act more as a plasticizer rather than stabilizing the polysaccharide network. This can be a result of their low molecular mass (1–1.8 kDa) [10,11]. The decrease in Young’s modulus (Table 3) shows that the films behave less rigidly with increasing addition of keratin hydrolysates, proving their plasticizing character. Thus, the interactions between polysaccharides and polypeptides decisively influence the mechanical properties of the membranes. Consequently, the mechanical properties of the composite materials can be controlled by the targeted addition of keratin hydrolysates.

### 3.6. Thermal Analysis of the Hybrid Films

**Figure 7:**
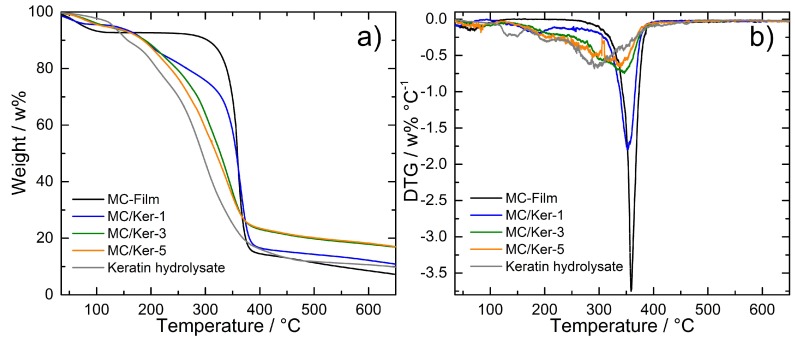
(**a**) Thermogravimetric analysis (TGA) and (**b**) Differential thermogravimetric curves (DTG) of keratin hydrolysates, methyl cellulose film (without plasticizer) and composite membranes (including 25 μL glycerol as plasticizer) with different methyl cellulose/keratin hydrolysate (MC/Ker) ratios.

In order to improve the mechanical properties as well as the water resistance of the composite materials, chemical crosslinking steps—preferably with non-toxic and environmentally friendly substances—are required. These usually involve a heat treatment to activate the chemical reaction. Therefore, knowledge about the thermal stability of the membranes is of great importance. This can be provided by TGA examinations on the materials. Dried keratin hydrolysates, a pure methyl cellulose film without further additives (MC-Film), and the composite membranes MC/Ker-1, MC/Ker-3, and MC/Ker-5—including 25 μL glycerol as plasticizer—were analyzed (Figure 7). The examined samples show a small weight loss up to 120 °C. This can be correlated to the loss of residual moisture. For the pure methyl cellulose, this weight loss amounts to 7.5 w% and is thereby significantly larger compared to the corresponding mass loss of pure keratin hydrolysate (3.5 w%). For the composite membranes, the first weight loss step amounts to 4.5–5 w%. This is due to the fact that the synthesized keratin polypeptides mostly consist of hydrophobic amino acids [11]. Thus, the water absorption ability of the membranes may drop in the presence of the keratin hydrolysates.

The decomposition of the pure keratin hydrolysates begins at approximately 130 °C and consists of several partly overlapping stages. In its differential thermogravimetric (DTG) curve—the first derivative of the TG-curve—beyond the evaporation of water (peak minima around 80 °C), further peak minima at 130 °C, 160 °C, and between 285 and 300 °C can be identified, marking the maximum degradation rate of these decomposition steps. Around 85 w% of the initial polypeptide mass (excluding sample humidity) decomposes during the heat treatment. Methyl cellulose, in contrast, decomposes in one defined mass loss step with its maximum degradation rate at 359 °C.

Beyond the discussed loss of residual water up to 120 °C, a second stage between 130 and 280 °C corresponding to the superimposed thermal decomposition of both, glycerol and keratin hydrolysates, is noticed for the composite films. A third weight loss stage observed from 280 to 450 °C results from the thermal decomposition of the remaining methyl cellulose-rich components of the membranes. This step becomes more prominent with increasing methyl cellulose fraction. For high amounts of embedded polypeptides, this weight loss step takes place over a larger temperature range, and the peaks in the DTG curves get broader. This suggests the superimposed degradation of several molecular species. For all hybrid films, the thermal stability is higher than the stability of pure keratin hydrolysates but obviously lower than that of neat methyl cellulose. Thus, a trend can be observed suggesting that the stability of the hybrid films can be improved with increasing methyl cellulose content. Not all films completely decompose. Between 10 w% for the methyl cellulose-rich films and 20 w% for the keratin hydrolysate-rich films remain in the crucible.

## 4. Conclusions

Feather keratin can be dissolved using superheated water; the amount of dissolved material strongly depends on temperature and duration of the heat treatment. With temperatures around 220 °C and 120 min dwell time, up to 76 w% of the initial goose feather material can be dissolved. The ordered structure of the proteins in the feathers is no longer present in the dried and homogenized keratin hydrolysates. WAXD patterns only display a broad hump at a diffraction angle 2*θ* around 19° instead of sharp protein diffraction peaks.

As the dried polypeptides form highly brittle layers, a supporting material is needed for the film casting. With methyl cellulose as matrix material, flexible composite membranes were casted from the solution. Materials with methyl cellulose to keratin hydrolysate ratios (MC/Ker) between 300/83.75 and 300/418.75 are produced without traces of macroscopic separation. The films are free from visible defects and exhibited a smooth surface. These novel bio-membranes are expected to combine both properties of proteins and polysaccharides. FTIR analysis of the films shows absorption peaks corresponding to methyl cellulose as well as amide bands of the polypeptides. WAXD analysis of the films proves the incorporation of the keratin hydrolysate molecules into the semi-crystalline methyl cellulose matrix. The mechanical properties are also found to depend on the amount of incorporated keratin hydrolysates. With an increasing fraction of hydrolysates in the films, Young’s modulus and offset yield strength decrease, while the ultimate elongation at break increases. Hence, the oligopeptides might act more like a plasticizer rather than stabilizing the matrix polymer network. This can be explained by the low molecular weight of the keratin hydrolysates.

The thermal stability of all hybrid films is higher than for pure keratin hydrolysates but lower compared to neat methyl cellulose films. This has to be taken into account for potential applications or subsequent processing steps such as crosslinking, which are necessary to improve the mechanical properties and the water resistance of the composite membranes. In order to maintain the “green” character of the methyl cellulose/keratin hydrolysate materials, it is important that the chemicals used for crosslinking are non-toxic and environmentally friendly.

## Figures and Tables

**Table 1:** Overview of the produced composite films.

Sample	V${}_{MC}$ ^1^	dwt${}_{MC}$	V${}_{Ker}$ ^2,3^	dwt${}_{Ker}$	MC/Ker Ratio in Dried Films
MC-Film	15 mL	300 mg	-	-	300/0
MC/Ker-1	15 mL	300 mg	0.5 mL	83.75 mg	300/83.75
MC/Ker-2	15 mL	300 mg	1 mL	167.5 mg	300/167.5
MC/Ker-3	15 mL	300 mg	1.5 mL	251.25 mg	300/251.25
MC/Ker-4	15 mL	300 mg	2 mL	335 mg	300/335
MC/Ker-5	15 mL	300 mg	2.5 mL	418.75 mg	300/418.75

^1^ 20 mg/mL; ^2^ polypeptide content: 250 mg/mL·67%; ^3^ process parameter: 250 mg/mL, 120 min, 220 °C.

**Table 2:** Amount of feathers dissolved by superheated water (w%)–calculated by Equation (1)—for different initial feather to water ratios, temperatures and dwell times.

Feathers	Yield at 180 °C			Yield at 200 °C			Yield at 220 °C		
	30 min	60 min	120 min	30 min	60 min	120 min	30 min	60 min	120 min
20 mg/mL	2.5%	14.3%	55.3%	4.2%	14.8%	63.5%	2.2%	45.8%	76.0%
50 mg/mL	3.8%	6.2%	46.1%	2.2%	7.2%	61.6%	2.6%	23.5%	56.4%
100 mg/mL	2.5%	4.9%	38.4%	2.9%	6.0%	54.8%	2.3%	35.1%	64.5%
250 mg/mL	1.6%	3.2%	45.4%	2.2%	5.9%	39.4%	2.9%	23.8%	67.3%

**Table 3:** Mechanical properties of hybrid films with different methyl cellulose/keratin hydrolysate (MC/Ker) ratios. An amount of 25 μL glycerol was added as plasticizer to the film forming solutions.

Sample	Young’s Modulus	Offset Yield Strength	Ultimate Elongation
MC/Ker-1	544.2 MPa ± 16.6 MPa	27.8 MPa ± 1.9 MPa	45.2% ± 5.9%
MC/Ker-2	474.0 MPa ± 66.3 MPa	25.2 MPa ± 2.4 MPa	48.7% ± 4.4%
MC/Ker-3	273.5 MPa ± 26.7 MPa	18.3 MPa ± 2.4 MPa	73.0% ± 5.4%
MC/Ker-4	168.5 MPa ± 20.3 MPa	12.7 MPa ± 0.5 MPa	80.6% ± 1.4%
MC/Ker-5	148.5 MPa ± 13.3 MPa	10.7 MPa ± 1.1 MPa	93.5% ± 2.2%

## Abbreviations

The following abbreviations are used in this manuscript:

MC	Methyl cellulose
Ker	Keratin hydrolysate
FTIR	Fourier transform infrared spectroscopy
WAXD	Wide angle X-ray diffraction
TGA	Thermogravimetric analysis
DTG	Differential thermogravimetric curve
